# Supplementary material for: Population genomics of two invasive mosquitoes (Aedes aegypti and Aedes albopictus) from the Indo-Pacific
Source: PLoS Negl Trop Dis. 2020 Jul 17;14(7):e0008463. doi: 10.1371/journal.pntd.0008463 (PMC7390453; doi:10.1371/journal.pntd.0008463)
Supplement: S1 Text — (DOCX) [file pntd.0008463.s001.docx]

This file is supplementary information for the publication:

“Population genomics of two invasive mosquitoes (*Aedes aegypti* and *Aedes albopictus*) from the Indo-Pacific”

Authors: Thomas L Schmidt, Jessica Chung, Ann-Christin Honnen, Andrew R Weeks, Ary A Hoffmann

**DNA extraction, SNP genotyping and filtering**

DNA was extracted from mosquitoes using either Qiagen DNeasy Blood & Tissue Kits (Qiagen, Hilden, Germany) or Roche High Pure™ PCR Template Preparation Kits (Roche Molecular Systems, Inc., Pleasanton, CA, USA), each with an RNase A treatment step. We applied the double digest restriction-site associated DNA sequencing (ddRADseq) protocol for *Ae. aegypti* developed by Rašić et al. (2014) to construct RAD libraries for *Ae. aegypti*, and used the protocol for *Ae. albopictus* adapted by Schmidt et al. (2017).

We performed initial digestions of 30 – 200 ng of genomic DNA, using 10 units each of MluCI and NlaIII restriction enzymes (New England Biolabs, Beverly MA, USA), NEB CutSmart buffer, and water. Digestions were run for 3 hours at 37 °C with no heat kill step, and the products were cleaned with paramagnetic beads. These were ligated to modified Illumina P1 and P2 adapters overnight at 16 °C with 1,000 units of T4 ligase (New England Biolabs, Beverly, MA, USA), followed by a 10-minute heat-deactivation step at 65 °C. We performed size selection with a Pippin-Prep 2% gel cassette (Sage Sciences, Beverly, MA) to retain DNA fragments of either 350 – 450 bp or 320 – 450 bp.

Final libraries were amplified by PCR, using 1 μL of size-selected DNA, 5 μL of Phusion High Fidelity 2× Master mix (New England Biolabs, Beverly MA, USA) and 2 μL of 10 μM standard Illumina P1 and P2 primers. These were run for 12 PCR cycles, then cleaned and concentrated using 0.8× paramagnetic beads. Each ddRAD library contained between 30 and 65 mosquitoes, and each was sequenced on a single sequencing lane using 100 bp chemistry. Libraries were sequenced paired-end at either the Australian Genome Research Facility (AGRF, Melbourne, Australia), the University of Melbourne (Pathology), or GeneWiz, Inc (Suzhou, China) on either a HiSeq 2500 or a HiSeq 4000 (Illumina, California, USA).

We used the Process_radtags program in Stacks v2.0 [3] to demultiplex sequence reads and trim the reads to 80 bp in length. Using a 15 bp sliding window, low quality reads were discarded if the average phred score dropped below 20. We used Bowtie v2.0 [4] to align reads to the *Ae. aegypti* [5] and *Ae. albopictus* [6] mitochondrial genome (mtDNA) assemblies, using --very-sensitive alignment settings. Reads that did not align to the mtDNA assemblies were aligned to the nuclear assemblies AaegL5 [5] and AaloF1 [7] to obtain nuclear genotypes for *Ae. aegypti* and *Ae. albopictus* respectively, using --very-sensitive alignment settings.

**Kin filtering**

We used the Stacks Ref_map program to build individual Stacks catalogs for each of the 16 *Ae. aegypti* and 20 *Ae. albopictus* populations, from which we called genotypes at RAD stacks at a 0.05 significance level. We generated VCF files for each catalog with the Stacks program Populations. SNPs were required to be present in ≥ 75% of the mosquitoes and have a minor allele count of > 2 (-r 0.75 –mac 2 --vcf). We used VCFtools [8] to thin the data so that no two SNPs were within 250 kbp of one another. Mosquitoes with ≥ 30% missing data were omitted from all further analyses.

We used SPAGeDi [9] to calculate Loiselle’s *k* [10] among mosquitoes, identifying pairs with putative first-degree relatedness (*k* ≥ 0.1875) and omitting related mosquitoes in order of missing data so that all remaining pairs had *k* < 0.1875. Finally, for populations with more than 18 mosquitoes, we omitted mosquitoes in order of missing data to reduce the number to 18.

**References**

1. Rašić G, Filipović I, Weeks AR, Hoffmann AA. Genome-wide SNPs lead to strong signals of geographic structure and relatedness patterns in the major arbovirus vector, *Aedes aegypti*. BMC Genomics. 2014;15(1):275.

2. Schmidt TLTL, Rašić G, Zhang D, Zheng X, Xi Z, Hoffmann AAA. Genome-wide SNPs reveal the drivers of gene flow in an urban population of the Asian Tiger Mosquito, *Aedes albopictus*. Lenhart A, editor. PLoS Negl Trop Dis. 2017;11(10):e0006009.

3. Catchen J, Hohenlohe PA, Bassham S, Amores A, Cresko WA. Stacks: an analysis tool set for population genomics. Mol Ecol. 2013;22(11):3124–40.

4. Langmead B, Salzberg SL. Fast gapped-read alignment with Bowtie 2. Nat Methods. 2012;9(4):357–9.

5. Matthews BJ, Dudchenko O, Kingan SB, Koren S, Antoshechkin I, Crawford JE, et al. Improved reference genome of *Aedes aegypti* informs arbovirus vector control. Nature. 2018;1.

6. Zhang H, Xing D, Wang G, Li C, Zhao T. Sequencing and analysis of the complete mitochondrial genome of *Aedes albopictus* (Diptera: Culicidae) in China. Mitochondrial DNA. 2016;27(4):2787–8.

7. Chen X-G, Jiang X, Gu J, Xu M, Wu Y, Deng Y, et al. Genome sequence of the Asian Tiger mosquito, *Aedes albopictus*, reveals insights into its biology, genetics, and evolution. Proc Natl Acad Sci. 2015;112(44):E5907–15.

8. Danecek P, Auton A, Abecasis G, Albers CA, Banks E, DePristo MA, et al. The variant call format and VCFtools. Bioinformatics. 2011;27(15):2156–8.

9. Hardy OJ, Vekemans X. SPAGeDi: a versatile computer program to analyse spatial genetic structure at the individual or population levels. Mol Ecol Notes. 2002;2(4):618–20.

10. Loiselle BA, Sork VL, Nason J, Graham C. Spatial genetic structure of a tropical understory shrub, *Psychotria officinalis* (Rubiaceae). Am J Bot. 1995;82(11):1420–5.
