# Supplementary figures and images for: Population genomics of two invasive mosquitoes (Aedes aegypti and Aedes albopictus) from the Indo-Pacific
Source: PLoS Negl Trop Dis. 2020 Jul 17;14(7):e0008463. doi: 10.1371/journal.pntd.0008463 (PMC7390453; doi:10.1371/journal.pntd.0008463)

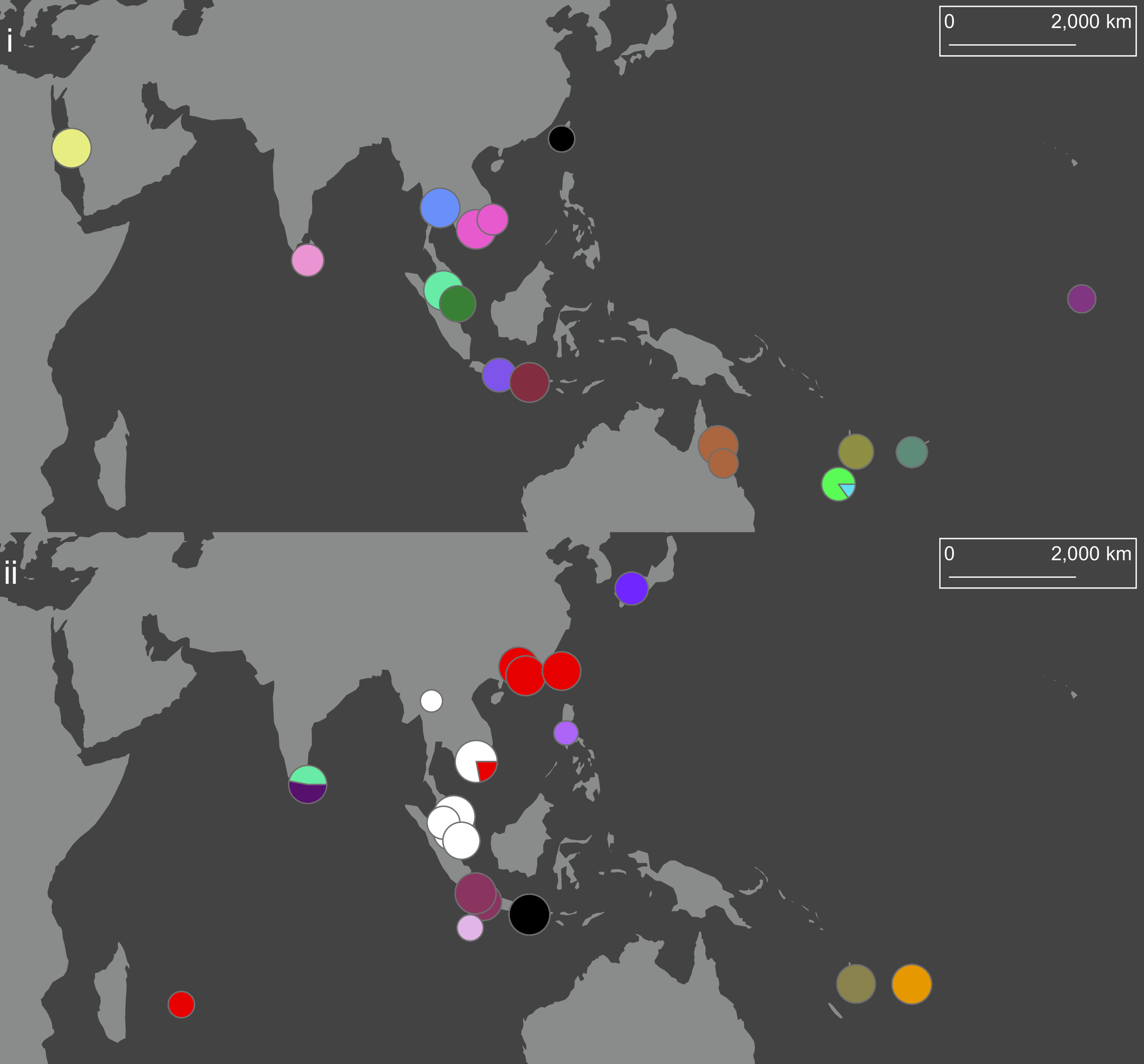

Supplement: S1 Fig — Colours indicate cluster membership. Circles are sized relative to population sample size. For Ae. aegypti, K = 15 was used, while K = 11 was used for Ae. albopictus. The map uses a Mollweide projection with a central meridian of 120°E. The map was produced in arcmap 10.5.1, using shapefiles made available by www.naturalearthdata.com. (TIF) [file pntd.0008463.s003.tif]
